# *ZmEIL2* positively regulates drought tolerance in maize by modulating stomatal movement, hormonal homeostasis, and antioxidant defense

**DOI:** 10.3389/fpls.2026.1946405

**Published:** 2026-09-18

**Authors:** Yue Li, Yanjun Zhang, Xusheng Qi, Zuowang Gou, Hongmei Wang, Xianfeng Yang, Jingfu Ma, Xingrong Wang

**Affiliations:** Institute of Crop, Gansu Academy of Agricultural Sciences/Key Laboratory of Crop Gene Resources and Germplasm Innovation in Northwest Cold and Arid Regions (Co-construction by Ministry and Province), Ministry of Agriculture and Rural Affairs, Lanzhou, China

**Keywords:** antioxidant defense, drought tolerance, hormonal homeostasis, stomatal movement, *Zea mays*, *ZmEIL2*

## Abstract

Drought stress severely constrains global maize productivity. Although ethylene (ETH)-responsive transcription factors play key roles in plant stress adaptation, the functional mechanism of *ZmEIL2* in drought tolerance remains poorly understood. Here, transgenic maize lines with *ZmEIL2* overexpressing (OE) and *ZmEIL2* knockout (KO) were generated and subjected to progressive drought stress followed by rehydration. Phenotypic analysis indicated that *ZmEIL2* OE lines exhibited improved plant survival and reduced leaf water loss, while KO lines exhibited displayed enhanced drought sensitivity. Physiological assessments demonstrated that *ZmEIL2* preserves leaf relative water content by regulating stomatal movement and mitigates drought-induced membrane damage and lipid peroxidation, which correlated with increased proline (Pro) accumulation. Furthermore, *ZmEIL2* modulated endogenous ETH and abscisic acid (ABA) levels under drought stress and enhanced the antioxidant defense system by increasing the activities of superoxide dismutase (SOD), peroxidase (POD) and ascorbate peroxidase (APX), thereby diminishing reactive oxygen species (ROS) accumulation, as evidenced by 3,3’-diaminobenzidine (DAB) and nitro blue tetrazolium (NBT) staining. We performed RNA sequencing (RNA-seq)on wild-type (WT), OE, and KO plants sampled at 0, 10, and 13 days of drought treatment and identified 120 core drought-responsive genes involved in stress perception, oxidoreductase activity, and metabolic reprogramming. Notably, 11 candidate genes, including *ZmSOD16*, *ZmPAL7*, *ZmHSP101*, *ZmHSP70*, *ZmGA2ox3*, and *ZmSMT1*, exhibited strictly opposite expression trends between OE and KO under normal conditions, suggesting their roles in antioxidant defense, protein protection, and hormone and carbohydrate metabolism. Collectively, these results demonstrate that *ZmEIL2* as a positive regulator of drought tolerance in maize via a multi-layered regulatory mechanism encompassing stomatal regulation, hormonal balance, membrane integrity, osmotic adjustment, and ROS scavenging. This work provides valuable candidate genes for the breeding of drought-resilient maize varieties.

## 1 Introduction

Maize (*Zea mays* L.) stands as one of the world’s most vital crops, serving as a cornerstone for food security, animal feed, and industrial raw materials (Lobell et al., 2014; Sheoran et al., 2022). However, its productivity is severely constrained by drought stress-the primary abiotic factor limiting global maize production (Sheoran et al., 2022). As climate change intensifies the frequency and severity of drought events, understanding the molecular basis of maize drought tolerance has become an urgent priority for sustainable agriculture.

Plants have evolved sophisticated adaptive strategies to cope with water deficit. At the physiological level, drought triggers stomatal closure to minimize transpirational water loss, alongside the accumulation of osmotic regulators such as proline (PRO) and the activation of enzymatic antioxidants including superoxide dismutase (SOD), peroxidase (POD), and ascorbate peroxidase (APX) to scavenge excess reactive oxygen species (ROS) (Zhao et al., 2005; Abou-Elwafa and Shehzad, 2018; Cruz De Carvalho, 2008). At the molecular level, these responses are orchestrated by intricate hormone signaling networks, with abscisic acid (ABA) and ethylene (ETH) serving as central players (Rhaman et al., 2024). ABA promotes the expression of stress-responsive genes encoding dehydrins and aquaporins, while ETH modulates diverse abiotic stress responses through its signaling cascade (Zhang and Dong, 2016; Beaudoin et al., 2000). Importantly, crosstalk between these two hormones fine-tunes the plant’s ability to balance growth and stress adaptation.

In the ETH signaling pathway, the EIN3/EIL (ETHYLENE-INSENSITIVE3/EIN3-LIKE) family of transcription factors functions as a critical node downstream of ETH perception, relaying signals from the upstream EIN2 component to activate the transcriptional expression of downstream target genes, including numerous ETH response factors (Iuchi et al., 2001; Ali et al., 2026; Alonso et al., 1999). Considerable progress has been made in characterizing EIN3/EIL functions in model species. In *Arabidopsis*, EIN3 and its homolog EIL1 act redundantly to govern nearly all ETH responses (Alonso et al., 1999). In rice, *OsEIL1* and *OsEIL2* have been shown to regulate ROS homeostasis and participate in abiotic stress responses, and both are recognized as master regulators of ETH signaling (Qiao et al., 2024). Moreover, a recent genome-wide identification of genes involved in stomatal closure under drought in maize identified *ZmEIL5* as one of twelve key candidate genes regulating stomatal aperture, highlighting the potential relevance of EIL family members to drought adaptation in this crop (Liu et al., 2025). Beyond the EIN3/EIL family, other transcription factor families—including AP2/ERF, MYB, and bZIP—have been extensively documented as prominent regulators within the maize drought response network (Ren et al., 2024; Liu Z. et al., 2026; Li W. et al., 2022).

Our research group has previously conducted the cloning, bioinformatics analysis, subcellular localization, and identification of stress expression patterns for the *ZmEIL2* gene in maize. It has been established that this gene can be significantly induced by various abiotic stresses, including drought, polyethylene glycol (PEG), ABA, and high salinity. Preliminary inferences suggest that *ZmEIL2* is involved in the stress response processes of maize (Li et al., 2023). However, the specific physiological effects of *ZmEIL2* on regulating drought tolerance, the transcriptional regulatory pathways, and the interactions between upstream and downstream molecules remain unclear.

Building on the aforementioned research background, this study utilized the maize inbred line B104 as the wild-type material and selected *ZmEIL2* overexpression strains (OE1, OE3) alongside knockout strains (KO3, KO4) for experimentation. By integrating phenotypic identification, physiological and biochemical indicator assessment, transcriptome sequencing, and quantitative real-time polymerase chain reaction (qRT-PCR) verification techniques, we systematically analyzed the physiological and molecular mechanisms underlying *ZmEIL2*-mediated drought responses in maize. The findings of this study offer candidate genes and theoretical insights for the molecular breeding of drought-resistant maize.

## 2 Results

### 2.1 *ZmEIL2* overexpression enhances seedling drought tolerance in maize

To evaluate the role of *ZmEIL2* in drought tolerance, we performed drought–rewatering phenotyping assays in two independent batches, with wild-type B104 (WT) included as a contemporaneous control in each batch. Under well-watered conditions, all transgenic lines showed comparable growth status relative to their matched WT controls, with no obvious phenotypic differences (**Figures 1A, B**). In the WT/OE experimental batch (lines OE1, OE3), after 14 d of water withholding, WT plants exhibited severe leaf wilting, whereas *ZmEIL2*-overexpression lines displayed markedly milder wilting symptoms (**Figure 1A**). Following 3 d of rewatering, OE1 and OE3 showed superior recovery capacity compared with the contemporaneous WT (**Figure 1A**), with survival rates increased by 1.1-fold and 1.5-fold, respectively (**Figure 1C**). In the independent WT/KO experimental batch (lines KO3, KO4), 12 d of drought treatment induced more pronounced wilting in knockout lines than in the matched WT control, manifested as severely curled leaves and chlorotic leaf tips (**Figure 1B**). After 3 d of rehydration, KO3 and KO4 exhibited limited recovery (**Figure 1B**) and significantly lower survival rates relative to WT (**Figure 1D**).

**Figure 1:**
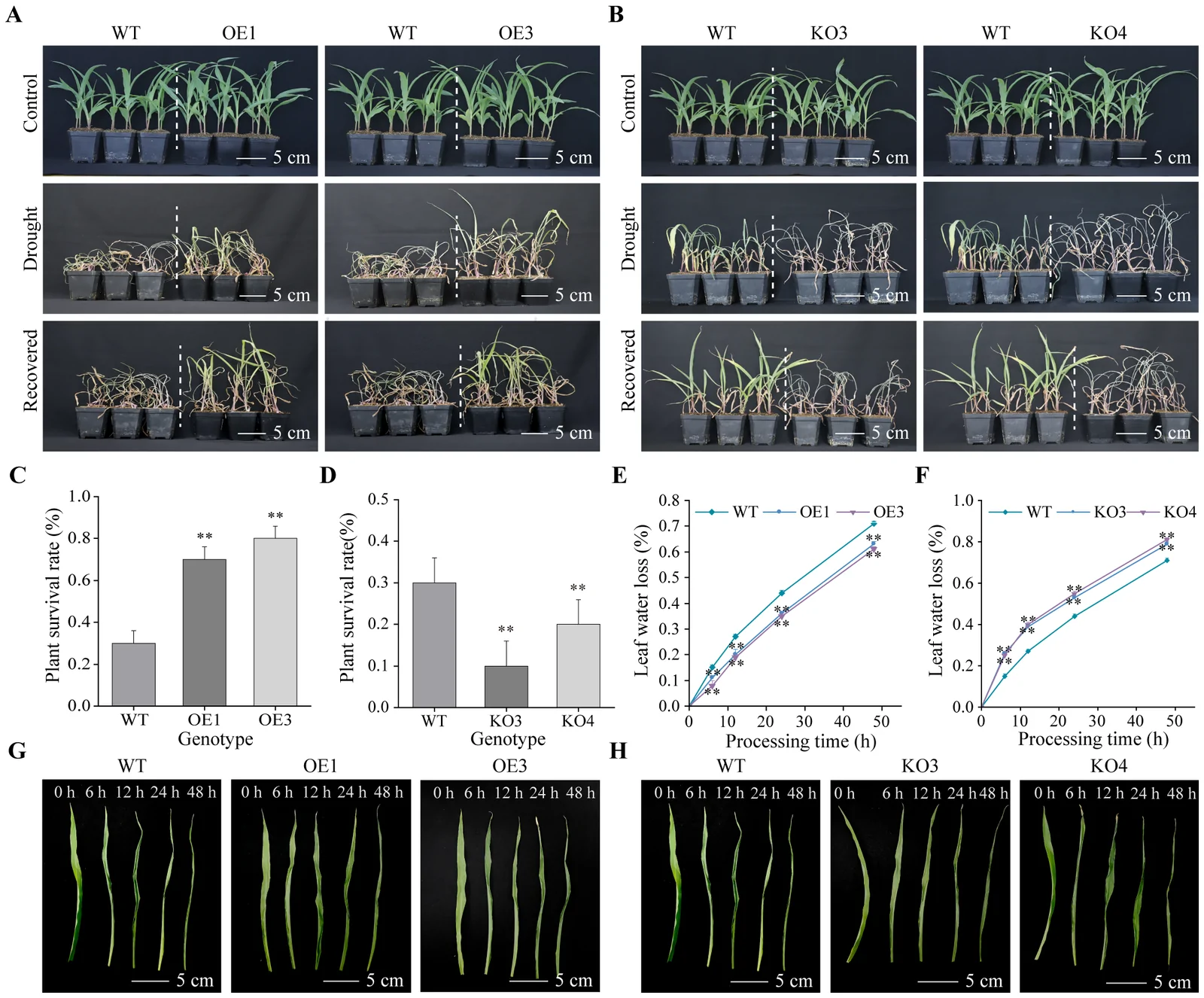
Overexpression of *ZmEIL2* enhances the drought tolerance of maize. **(A)**, Phenotypic analysis of WT and *ZmEIL2****-***OE for drought tolerance **(B)**, Phenotypic analysis of WT and *ZmEIL2****-***KO for drought tolerance **(C)**, survival rates of WT and *ZmEIL2****-***OE **(D)**, survival rates of WT and *ZmEIL2****-***KO **(E)**, Leaf water loss of WT and *ZmEIL2****-***OE **(F)**, Leaf water loss of WT and *ZmEIL2****-***KO **(G)**, Phenotype of water loss in WT and *ZmEIL2*-OE plants **(H)**, Phenotype of water loss in WT and *ZmEIL2****-***KO. The data were presented as means ± SD from three independent biological replicates. The significance analysis was performed by one-way ANOVA (Duncan test) (*p < 0.05, **p < 0.01).

In addition to evaluating the overall performance of the entire plant, we assessed the water retention capacity of the leaves through *in vitro* experiments measuring leaf water loss. During the dehydration treatment period, which ranged from 6 h to 48 h, the water loss rate of isolated leaves from the overexpressing strains OE1 and OE3 was significantly lower than that of the wild type (**Figure 1E**). Specifically, compared to the wild type, the water loss rates of OE1 at 6 h, 12 h, 24 h, and 48 h decreased by 27%, 26%, 19%, and 11%, respectively, while those of OE3 decreased by 44%, 30%, 22%, and 14%, respectively (**Figure 1E**). In contrast, the water loss rates of the knockout lines KO3 and KO4 were significantly increased (**Figure 1F**). Compared to the wild type, the water loss rates of KO3 increased by 64%, 48%, 23%, and 14% at 6 h, 12 h, 24 h, and 48 h, respectively, while those of KO4 increased by 73%, 44%, 20%, and 11% at the corresponding time points (**Figure 1F**). Under non-stress conditions, no significant abnormalities were observed in the leaf morphology of any strain (**Figures 1G, H**). However, following 48 h of continuous dehydration treatment, the leaves of the overexpressing lines OE1 and OE3 exhibited only slight curling compared to the wild type (**Figure 1G**), whereas the leaves of the knockout lines KO3 and KO4 displayed more pronounced curling and wilting (**Figure 1H**). These results suggest that the overexpression of *ZmEIL2* positively influences the drought tolerance of maize seedlings. These results indicated that *ZmEIL2* overexpression positively enhances drought tolerance of maize seedlings.

### 2.2 *ZmEIL2* modulates stomatal movement and leaf water retention

Leaf relative water content (RWC) serves as an indicator of water status and drought tolerance in plants, defined as the ratio of actual water content to the maximum saturated water content of leaves. Leaf RWC is closely linked to plant water use efficiency, with cellular water potential exerting a greater influence on plant growth under water deficit than stomatal conductance. We assessed leaf RWC in WT, *ZmEIL2*-OE, and *ZmEIL2*-KO maize plants under normal conditions, as well as following 10 d and 13 d of natural drought treatment (**Figures 2A, B**). Under well-watered conditions, RWC did not differ significantly among the genotypes (**Figures 2A, B**). However, after drought stress, OE1 and OE3 maintained significantly higher leaf RWC compared to WT (**Figure 2A**). Specifically, the RWC of OE1 increased by 6.70%, 6.83%, 9.87%, and 13.73%, while that of OE3 increased by 8.04%, 9.28%, 11.72%, and 17.54% at various measurement points (**Figure 2A**). In contrast, KO3 and KO4 exhibited markedly lower RWC relative to WT (**Figure 2B**).

**Figure 2:**
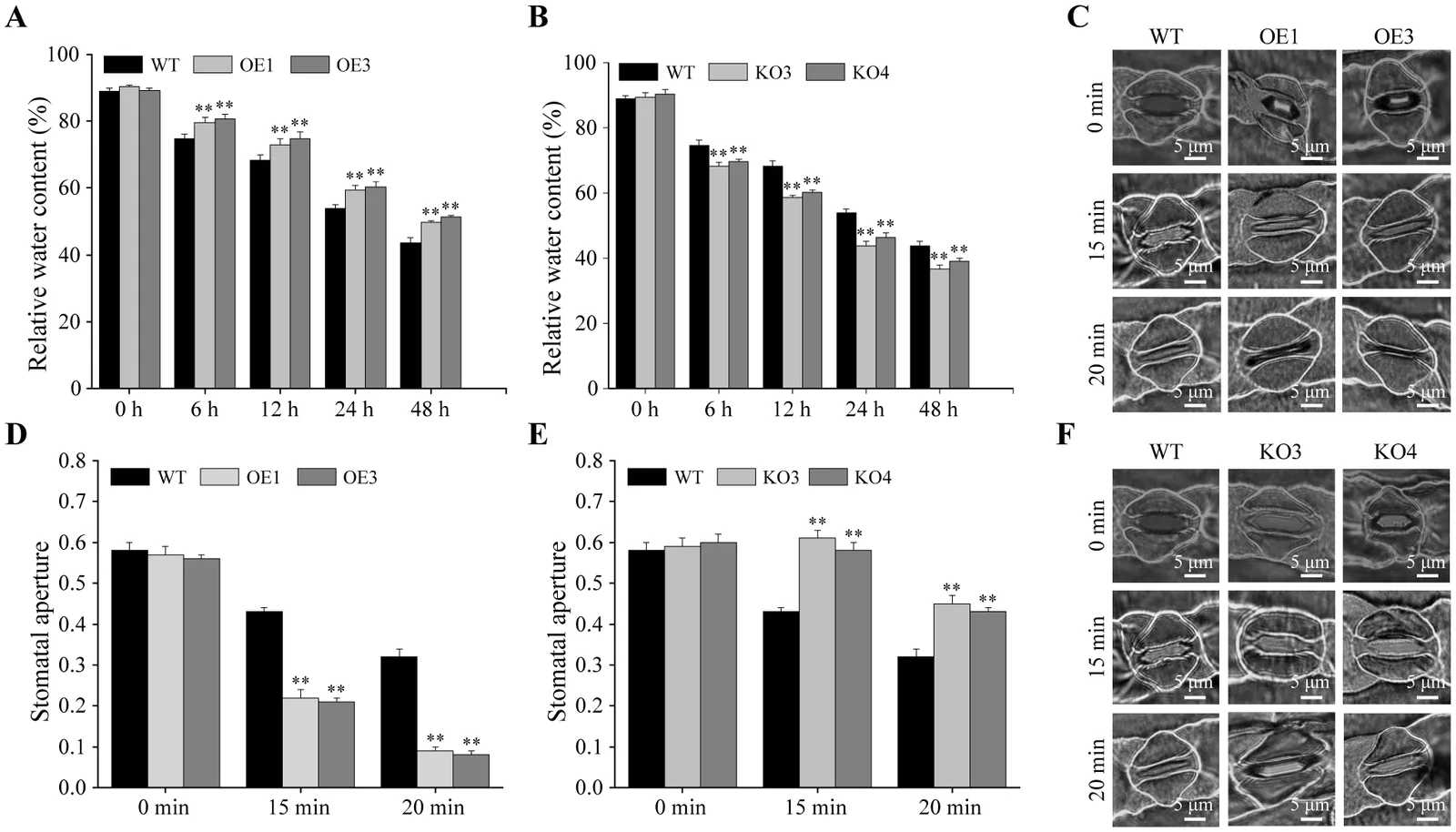
Physiological and stomatal phenotypic analysis of WT, *ZmEIL2*-OE and *ZmEIL2*-KO maize seedlings under drought treatment. **(A)**, RWC of WT and *ZmEIL2-*OE leaves **(B)**, RWC of WT and *ZmEIL2-*KO leaves **(C)**, Micrographs showing stomatal aperture of WT and *ZmEIL2-*OE leaves (20× magnification) **(D)**, Statistical quantification of stomatal aperture in WT and *ZmEIL2-*OE leaves **(E)**, Statistical quantification of stomatal aperture in WT and *ZmEIL2-*KO leaves **(F)**, Micrographs showing stomatal aperture of WT and *ZmEIL2-*KO leaves (20× magnification). The data were the average (± SD) of three independent biological replicates. The significance analysis was performed by one-way ANOVA (Duncan test) (*p < 0.05, **p < 0.01).

Stomata are critical structures for plant adaptation to drought, with transpirational water loss primarily regulated by stomatal dynamics. The rate of stomatal closure is an essential trait for minimizing water loss and enhancing drought resistance. To determine whether *ZmEIL2* influences water loss by regulating stomatal closure, we compared stomatal closure rates among various genotypes (**Figures 2D, E**). At 0 min post-dehydration, the extent of stomatal closure was comparable across WT, *ZmEIL2*-OE, and *ZmEIL2*-KO lines. Between 15 min and 20 min of dehydration, OE1 and OE3 exhibited significantly faster stomatal closure compared to WT (**Figures 2C, D**). In contrast, stomatal closure was markedly delayed in KO3 and KO4 (**Figures 2E, F**). Relative to WT, the degree of stomatal opening in KO3 increased by 42% and 40%, while KO4 showed increases of 36% and 34% (**Figure 2E**). In conclusion, *ZmEIL2* enhances leaf RWC by facilitating rapid stomatal closure under drought stress, thereby effectively reducing water loss and bolstering the drought resistance of maize plants.

### 2.3 *ZmEIL2* regulates endogenous hormone accumulation under drought stress

ABA functions as a fundamental stress-responsive hormone in plants. In response to drought conditions, endogenous ABA accumulation is stimulated, activating downstream signaling pathways, promoting the expression of drought-responsive genes, and triggering stomatal closure, which ultimately reduces transpirational water loss (Feng et al., 2025). To investigate whether *ZmEIL2* influences drought tolerance through the regulation of ABA and ETH biosynthesis, we measured the levels of these two hormones in WT, OE, and KO lines leaves under normal conditions, as well as after 10 d and 13 d of drought treatment (**Figures 3A–D**). Under well-watered conditions, no significant differences in ABA and ETH levels were observed among the genotypes. However, drought stress markedly affected endogenous hormone levels in a genotype-dependent manner. Drought treatment significantly enhanced ABA accumulation in *ZmEIL2*-OE lines compared to wild-type (WT) plants. The ABA levels in OE1 increased by 11.72% and 23.91%, while those in OE3 rose by 18.52% and 26.08% at the two drought time points (**Figure 3A**). In contrast, *ZmEIL2*-KO lines (KO3 and KO4) displayed markedly lower ABA levels than WT under drought stress (**Figure 3B**). Drought-induced ETH accumulation was consistently positively regulated by *ZmEIL2*. Compared to WT, the OE3 line exhibited significantly higher ETH production, with increases of 18.44% and 71.06% after 10 d and 13 d of drought, respectively (**Figure 3C**). In contrast, the KO4 line demonstrated a significant reduction in ETH release under drought conditions (**Figure 3D**).

**Figure 3:**
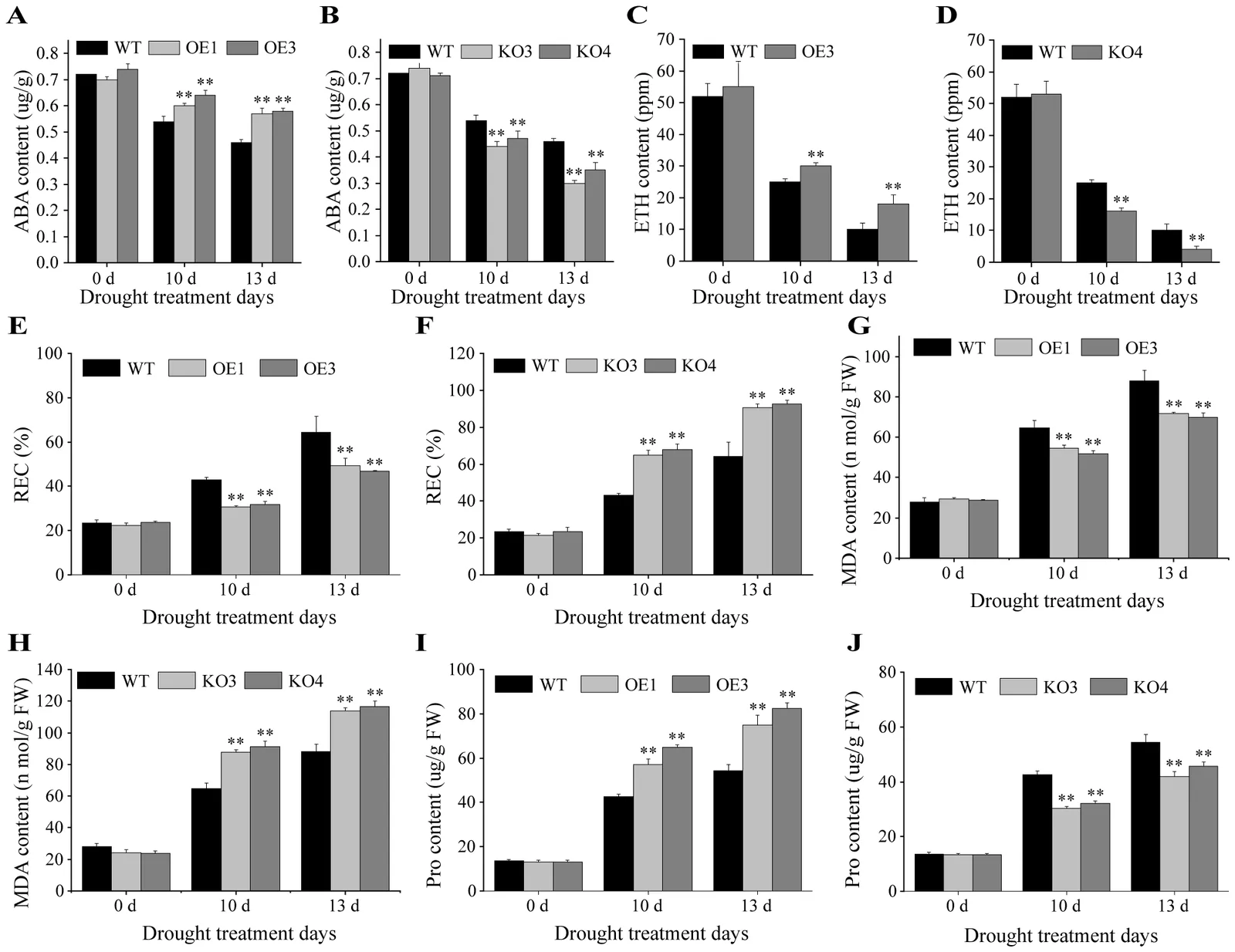
Determination of endogenous hormone levels and physiological stress-related indicators in WT, *ZmEIL2*-OE and *ZmEIL2*-KO maize under drought stress. **(A)**, ABA contents in WT and *ZmEIL2-*OE leaves **(B)**, ABA contents in WT and *ZmEIL2-*KO leaves **(C)**, ETH contents in WT and *ZmEIL2-*OE leaves **(D)**, ETH contents in WT and *ZmEIL2-*OE leaves **(E)**, REC of WT and *ZmEIL2-*OE leaves **(F)**, REC of WT and *ZmEIL2-*KO leaves **(G)**, MDA contents in WT and *ZmEIL2*-OE leaves **(H)**, MDA contents in WT and *ZmEIL2*-KO leaves **(I)**, Pro of WT and *ZmEIL2-*OE leaves **(J)**, Pro of WT and *ZmEIL2-*KO leaves. The data were the average (± SD) of three independent experiments. The significance analysis was performed by one-way ANOVA (Duncan test) (*p < 0.05, **p < 0.01).

Collectively, these findings suggest that *ZmEIL2* positively influences drought tolerance by enhancing the accumulation of ABA and ETH. This process promotes stomatal closure, reduces water loss, and ultimately improves drought adaptation in maize.

### 2.4 *ZmEIL2* alleviates cell membrane damage and regulates osmotic adjustment substances

Relative electrical conductivity (REC) and malondialdehyde (MDA) serve as critical physiological indicators for assessing cell membrane damage due to osmotic stress in plants (Zhang et al., 2022). To elucidate the regulatory role of *ZmEIL2* in drought-induced membrane injury, we measured REC and MDA levels in WT, OE, and KO maize leaves under well-watered conditions and following natural drought treatment for 10 d and 13 d. Under normal growth conditions, no significant differences in REC and MDA levels were detected among the genotypes. However, drought stress significantly altered their accumulation patterns (**Figures 3E–H**). Under drought conditions, *ZmEIL2*-OE lines (OE1 and OE3) exhibited markedly lower REC values compared to WT (**Figure 3E**). In contrast, *ZmEIL2*-KO lines (KO3 and KO4) displayed significantly increased REC levels, with KO3 showing increases of 58% and 44%, and KO4 showing increases of 51% and 41% (**Figure 3H**). Corresponding to the changes in REC, MDA contents exhibited similar variation trends. Drought treatment significantly reduced MDA accumulation in OE1 and OE3 relative to WT (**Figure 3G**), whereas KO3 and KO4 demonstrated substantially higher MDA levels, with KO3 increasing by 41.2% and 32.6%, and KO4 increasing by 35.6% and 29.5% under drought stress (**Figure 3H**).

Pro serves as a crucial osmoprotectant that accumulates dynamically in response to drought and other abiotic stresses, thereby maintaining cellular osmotic balance, protecting cellular structure, and enhancing plant adaptability to stress (Khan et al., 2025). To further elucidate the role of *ZmEIL2* in osmotic regulation, Pro levels were measured in various genotypes under normal conditions and following 10 d and 13 d of natural drought treatment (**Figures 3I, J**). The results indicated that Pro concentrations were similar among WT, OE, and KO lines under well-watered conditions. In contrast, under drought stress, OE1 and OE3 exhibited significantly higher Pro levels than WT, with OE1 increasing by 34% and 38% and OE3 by 52% and 51% at the two drought time points (**Figure 3I**). Conversely, the loss of function of *ZmEIL2* markedly diminished Pro accumulation in KO3 and KO4 leaves under drought conditions (**Figure 3J**). These findings suggest that *ZmEIL2* enhances the osmotic regulatory capacity of cells, preserves the structural integrity of cell membranes, and mitigates cellular damage induced by drought stress through the promotion of Pro accumulation, an osmotic regulatory compound.

### 2.5 *ZmEIL2* activates antioxidant defense system to reduce ROS accumulation

The accumulation of ROS due to drought is the primary cause of oxidative damage (Sun et al., 2023; Liu R. et al., 2026). This oxidative damage represents a fundamental mechanism through which drought stress inflicts harm on plants. The 3,3’-diaminobenzidine (DAB) staining intensity and contents of H_2_O_2_ were determined under normal growth conditions, and after 10 d and 13 d of natural drought treatment. Under well-watered conditions, no obvious differences in DAB staining and H_2_O_2_ accumulation were detected among WT, *ZmEIL2*-OE and *ZmEIL2*-KO plants (**Figures 4A–C**). Upon drought stress, leaves of *ZmEIL2*-OE lines (OE1, OE3) exhibited weaker DAB staining compared with WT, whereas *ZmEIL2*-KO lines (KO3, KO4) showed much deeper staining (**Figure 4A**). Consistent with the staining results, quantitative assays revealed significantly lower H_2_O_2_ concentrations in *ZmEIL2*-OE leaves under drought. Relative to WT, H_2_O_2_ levels increased by only 24.12% and 24.76% in OE1, and 34.80% and 28.90% in OE3 after drought treatment (**Figure 4B**). In contrast, *ZmEIL2*-KO lines accumulated markedly higher H_2_O_2_ than WT (**Figure 4A**). These data indicated that overexpression of *ZmEIL2* alleviates drought-triggered H_2_O_2_ overaccumulation by accelerating ROS scavenging capacity, thereby mitigating oxidative injury.

**Figure 4:**
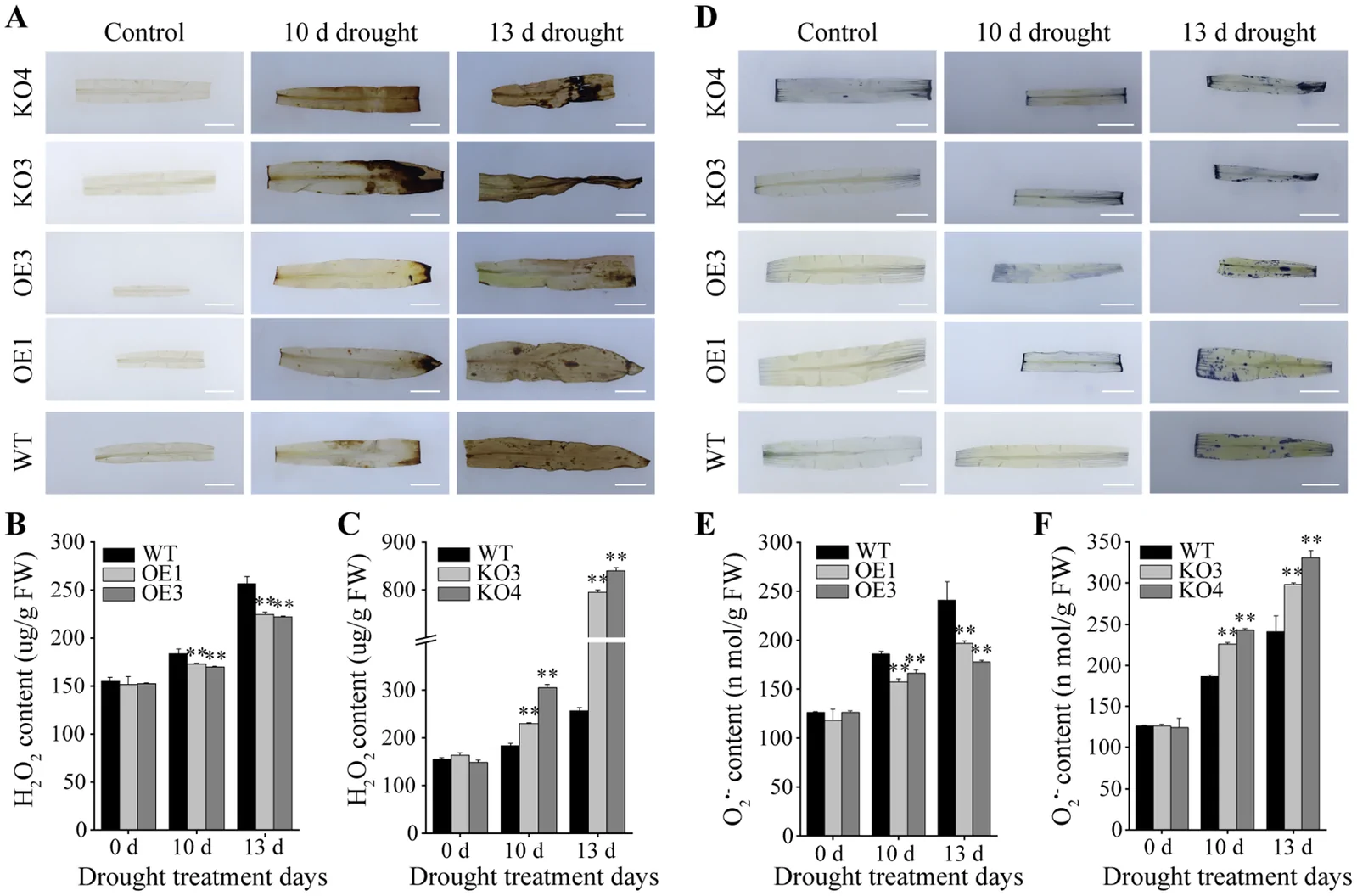
Histochemical staining and quantitative detection of reactive oxygen species in WT, *ZmEIL2*-OE and *ZmEIL2*-KO maize leaves under drought stress. **(A)**, DAB staining of leaf tissues from WT, *ZmEIL2*-OE and *ZmEIL2*-KO **(B)**, H_2_O_2_ contents in WT and *ZmEIL2*-OE leaves **(C)**, H_2_O_2_ contents in WT and *ZmEIL2*-KO leaves **(D)**, NBT staining of leaf tissues from WT, *ZmEIL2*-OE and *ZmEIL2*-KO **(E)**, O_2_^-^ production rate in WT and *ZmEIL2-*OE leaves **(F)**, O_2_^-^ production rate in WT and *ZmEIL2-*KO leaves. The data were the average (± SD) of three independent experiments. The scale A and D is 1 cm. The significance analysis was performed by one-way ANOVA (Duncan test) (*p < 0.05, **p < 0.01).

Nitro blue tetrazolium (NBT) staining and superoxide anion (O_2_^-^) production rate were further measured to assess superoxide accumulation under normal and 10/13 d natural drought treatments. Under well-watered conditions, NBT staining intensity and O_2_^-^ generation rates were comparable across WT, *ZmEIL2*-OE and *ZmEIL2*-KO genotypes (**Figures 4D–F**). After drought exposure, leaf NBT staining was lighter in OE1 and OE3, while KO3 and KO4 displayed intense staining (**Figure 4D**). Quantification confirmed that O_2_^-^ production rates were significantly lower in *ZmEIL2*-OE lines than in WT (**Figure 4E**). Conversely, O_2_^-^ levels were drastically elevated in knockout lines: KO3 showed 21.29% and 24.10% increases, and KO4 showed 30.24% and 37.40% increases relative to WT (**Figure 4F**). Collectively, these findings demonstrate that functional *ZmEIL2* suppresses drought-induced superoxide anion burst, which contributes to enhanced drought tolerance in maize.

The antioxidant defense system is essential for scavenging excess ROS generated by various abiotic stresses (Liu et al., 2026; Zhang et al., 2025). To investigate the effect of *ZmEIL2* on antioxidant defense enzymes in maize under drought stress, we examined the activities of three key enzymes: As shown in **Figures 5A–F**, the activities of SOD, POD and APX exhibited no obvious differences among wild-type (WT), *ZmEIL2*-OE and *ZmEIL2*-KO maize leaves under well-watered conditions. After natural drought treatment, SOD activity was significantly higher in *ZmEIL2*-OE lines (OE1, OE3) than in WT. Relative to WT, SOD activity increased by 4.84% and 18.50% in OE1, and 5.00% and 19.95% in OE3 (**Figure 5A**). By contrast, *ZmEIL2*-KO lines (KO3, KO4) displayed markedly lower SOD activity (**Figure 5B**).

**Figure 5:**
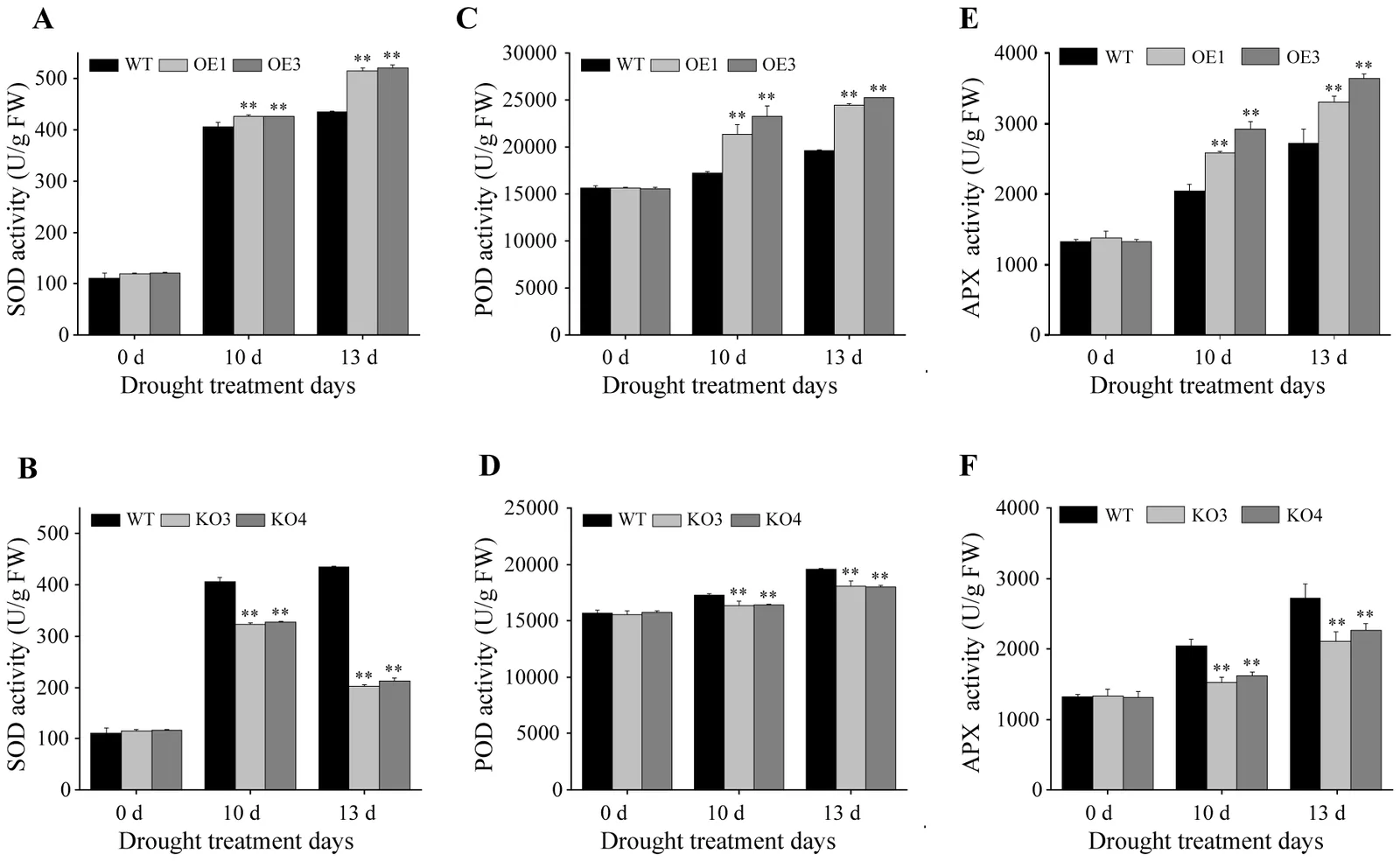
Determination of antioxidant enzyme activities in WT, *ZmEIL2*-OE and *ZmEIL2*-KO maize leaves under drought stress. **(A)**, SOD activities of WT and *ZmEIL2*-OE leaves **(B)**, SOD activities of WT and *ZmEIL2*-OE leaves **(C)**, POD activities of WT and *ZmEIL2*-OE leaves **(D)**, POD activities of WT and *ZmEIL2*-KO **(E)**, APX activities of WT and *ZmEIL2-*OE leaves **(F)**, APX activities of WT and *ZmEIL2-*KO leaves. The data were the average (± SD) of three independent experiments. The significance analysis was performed by one-way ANOVA (Duncan test) (*p < 0.05, **p < 0.01).

Consistent with the variation trend of SOD, POD activity was significantly elevated in OE1 and OE3 under drought. POD activity rose by 24.12% and 24.76% in OE1, and 34.80% and 28.90% in OE3 compared with WT (**Figure 5C**), while knockout lines KO3 and KO4 possessed remarkably lower POD activity (**Figure 5D**).

APX activity shared an identical changing pattern with SOD and POD. Drought stress induced significant increases in APX activity in OE1 and OE3: APX activity was enhanced by 26.34% and 21.21% in OE1, and 42.91% and 33.73% in OE3 (**Figure 5E**). However, APX activity was significantly suppressed in KO3 and KO4 leaves (**Figure 5F**).

Collectively, these results demonstrate that overexpression of *ZmEIL2* substantially elevates the activities of key antioxidant enzymes (SOD, POD and APX), accelerates intracellular ROS scavenging, and ultimately alleviates drought-induced oxidative damage in maize.

Taken together, these results demonstrate that *ZmEIL2* enhances the tolerance of maize plants to drought stress by inducing the activities of antioxidant defense enzymes and the accumulation of Pro and alleviating ROS level.

### 2.6 Transcriptome sequencing and functional analysis of differentially expressed genes

#### 2.6.1 Quality analysis of RNA-seq sequencing results

A total of 27 cDNA libraries were constructed from leaf samples of WT, *ZmEIL2*-OE, and *ZmEIL2*-KO maize lines collected at 0 d, 10 d, and 13 d following drought treatment. All libraries were sequenced on the Illumina HiSeq 6000 platform. Raw reads generated from sequencing were subjected to quality control filtering to remove reads containing adapters, excessive ambiguous N bases and low-quality sequences. The proportion of clean reads across all samples ranged from 94.55% to 98.06%. Meanwhile, the Q20 value of each sample exceeded 98.06%, and the Q30 value was higher than 92.56%, demonstrating reliable sequencing quality suitable for subsequent transcriptomic analysis. Detailed filtering statistics of sequencing data are summarized in **Supplementary Table 1**.

Clean reads were aligned to the maize reference genome using HISAT2 software, and the mapping statistics of all samples were summarized in **Supplementary Table 2**. The total mapping rates of all samples ranged from 85.95% to 91.01%, while the uniquely mapped reads accounted for 82.55%–87.89% of total clean reads, indicating high-quality sequencing data suitable for subsequent transcriptomic analysis.

We calculated Pearson correlation coefficients based on gene expression levels for every pair of samples and visualized the results using a correlation heatmap (**Supplementary Figure 2**). High correlation coefficients were observed among biological replicates, indicating a strong consistency in gene expression profiles within each group and providing reliable transcriptome data for subsequent analyses.

#### 2.6.2 Identification of differentially expressed genes under drought stress

Differentially expressed genes (DEGs) were identified using the thresholds of log_2_(FC) ≥ 1 and FDR < 0.05. In pairwise comparisons between WT and *ZmEIL2*-OE, we identified 1,792, 2,018, and 1,710 up-regulated differentially expressed genes (DEGs), along with 2,363, 633, and 2,972 down-regulated DEGs under 0 d, 10 d, and 13 d drought treatments, respectively (**Figure 6A**). In the WT versus *ZmEIL2*-KO comparison, we detected 329, 3,315, and 5,530 up-regulated DEGs, as well as 795, 2,240, and 5,872 down-regulated DEGs across the three time points (**Figure 6A**). Notable quantitative variations in DEGs were observed among the different treatment groups, indicating that the expression level of *ZmEIL2* significantly alters the transcriptional response of maize to drought stress in a time-dependent manner.(**Figure 6A**).

**Figure 6:**
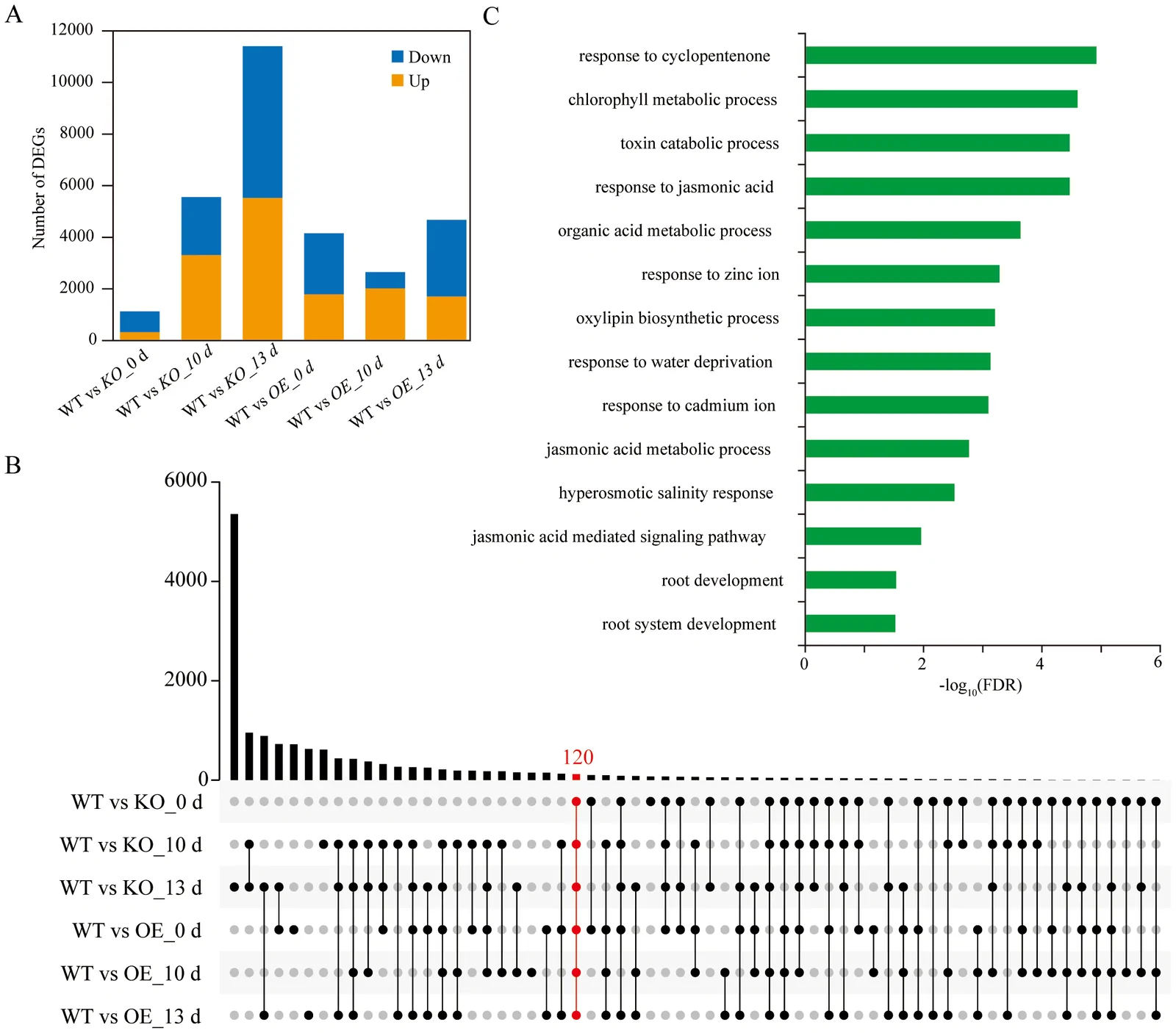
Statistics and distribution of DEGs among six genotype–drought treatment comparison groups. **(A)**, Quantification of up- and down-regulated DEGs for each comparison **(B)**, Dot plot displaying the distribution of total DEGs and 120 universal shared DEGs across all six groups. **(C)**, GO annotation of DEGs common to all six groups.

#### 2.6.3 Expression characteristics of drought stress response genes regulated by *ZmEIL2*

In this study, six comparative combinations were established: WT vs KO_0 d, and, WT vs KO_10 d, WT vs KO_13 d, WT vs OE_0 d, WT vs OE_10 d, and WT vs OE_13 d. An intersection analysis was performed on the differentially expressed genes across these combinations. The results from the Venn diagram (**Figure 6B**). revealed that a total of 120 common differentially expressed genes were identified among the six treatment groups (**Figure 6B**; **Supplementary Table 3**). This finding indicates that these genes consistently responded to drought stress across varying degrees of drought and genotypes, thereby categorizing them as core drought response genes.

Gene ontology (GO) biological process enrichment analysis (**Figure 6C**) revealed that this core gene set was significantly enriched in categories such as jasmonic acid response, cyclopentanone response, chlorophyll metabolism, toxin catabolism, and organic acid metabolism. These findings suggest that *ZmEIL2* can synergistically regulate multiple pathways, including hormone signaling, oxidative detoxification, photosynthetic homeostasis, and osmotic metabolism, thereby participating in the response to drought stress in maize.

Further analysis of the expression pattern of the core gene reveals two distinct regulatory characteristics between OE and KO strains. The first category of genes demonstrates an upregulation trend in both OE and KO lines under moderate drought conditions. This group includes the AP2/ERF family transcription factors *ZmERF115* (*Zm00001d016616*) and *ZmERF112* (*Zm00001d021089*), the heat shock transcription factor *ZmHSF4* (*Zm00001d020714*), the GT-2-like transcription factor *ZmGT-2-1* (*Zm00001d017420*), as well as key genes involved in jasmonic acid synthesis, such as *ZmAOS1* (*Zm00001d048021*), *ZmOPR2* (*Zm00001d044906*), and the antioxidant detoxification gene *ZmGSTU6* (*Zm00001d043795*). Notably, the upregulation of most genes intensifies under severe drought conditions in KO lines. It is speculated that these genes are indirectly regulated by *ZmEIL2* or that a feedback inhibition mechanism operates under drought stress. The second category of genes exhibits a clear positive regulatory pattern. The bZIP family transcription factor *ZmbZIP25* (*Zm00001d030995*) is significantly upregulated (log_2_FC=1.85) in the OE line under severe drought, whereas it is markedly downregulated (log_2_FC=-7.12) in the KO line. This indicates that *ZmbZIP25* is positively regulated by *ZmEIL2* during severe drought, potentially serving as a downstream mediator of the ABA signaling pathway to confer drought resistance.

In conclusion, the distinct expression characteristics of the 120 core drought response genes illustrate the multi-level and complex nature of the *ZmEIL2* regulatory network. This finding offers essential candidate targets for further investigation into the molecular mechanisms underlying drought resistance.

#### 2.6.4 Screening of candidate genes for reverse expression under normal conditions, functional annotation and verification of transcriptome data by qRT-PCR

To identify core regulatory genes exhibiting divergent expression patterns between WT and KO and OE strains under 0 d, this study conducted an intersection analysis of two sets of differentially expressed genes. The analysis revealed that the WT vs KO contained a total of 795 down-regulated differentially expressed genes, while WT vs OE included 1,792 upregulated differentially expressed genes. Ultimately, 11 genes demonstrating a consistent reverse expression pattern between the two groups were identified (**Supplementary Figure 3**). Based on functional annotations from the MapMan database (**Supplementary Table 4**), this set of candidate genes comprises the superoxide dismutase-encoding gene *ZmSOD16*, the FCS-type zinc finger protein gene *ZmFLZ15*, the phenylalanine ammonia-lyase gene *ZmPAL7*, the heat shock protein-encoding genes *ZmMS42* (which encodes HSP101) and *ZmHSP1* (which encodes HSP70), the α-amylase-encoding gene, the gibberellin 2-oxidase gene *ZmGA2OX3*, and the sterol methyltransferase gene *ZmSMT1*. These genes are involved in antioxidant defense, protein protection, hormone metabolism, and carbohydrate metabolism pathways, making them highly reliable candidates for drought response. They may serve as valuable targets for genetic engineering aimed at enhancing drought resistance in maize and for molecular marker-assisted breeding.

In this study, eight representative core genes were selected for further analysis. qRT-PCR was conducted to validate the reliability of the transcriptome data across six control combinations. These included comparisons of WT samples subjected to drought treatment for 0 d, 10 d, and 13 d with both OE and KO lines (**Figures 7A–F**). Linear regression analysis revealed that the coefficient of determination R² for the six data sets ranged from 0.870 to 0.913. This finding indicates a high level of consistency between the quantitative results of gene expression obtained from the two detection methods, thereby confirming the authenticity and reliability of the differential gene expression profiles identified through transcriptome sequencing.

**Figure 7:**
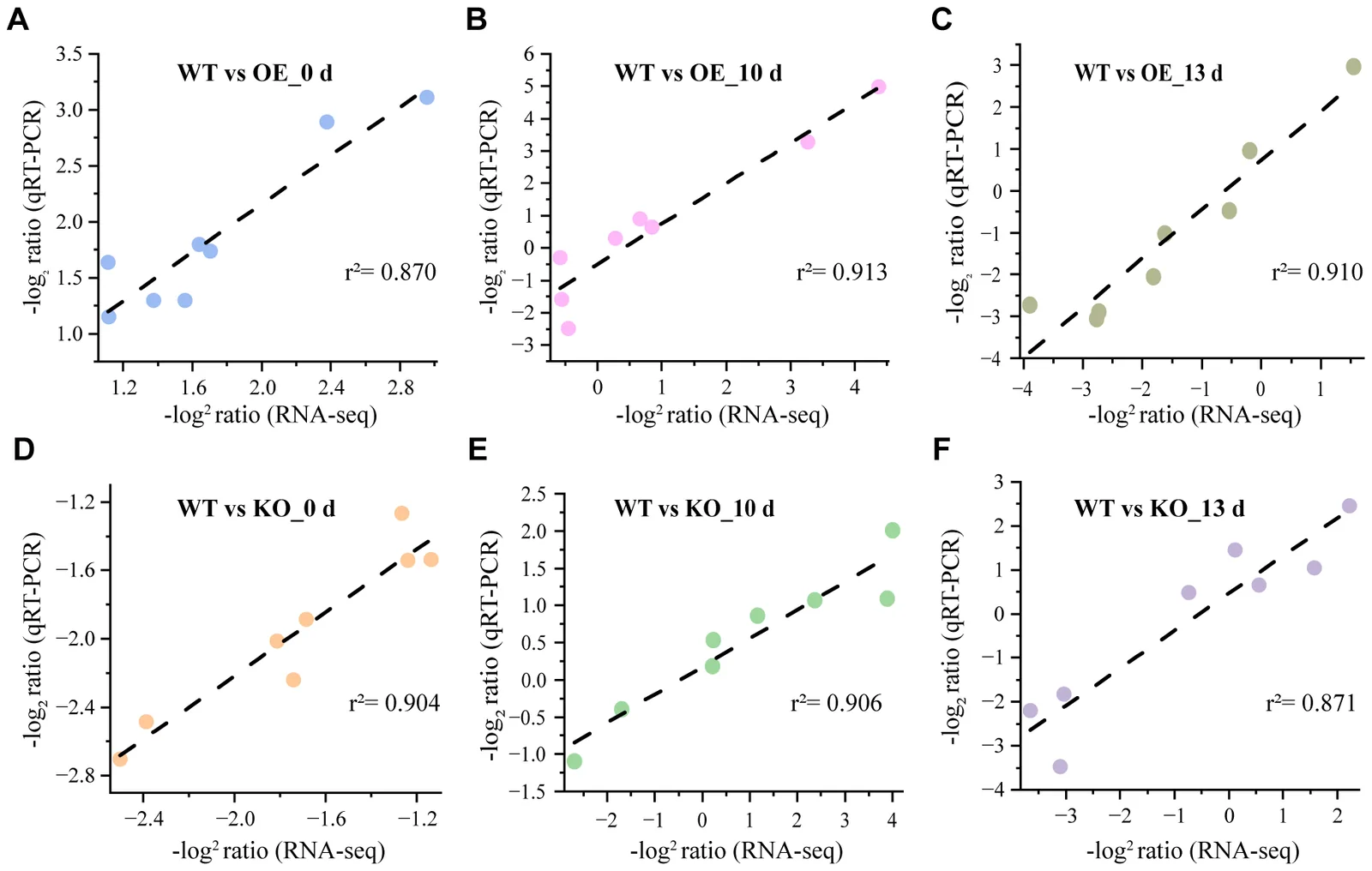
Correlation between gene expression fold changes measured by RNA-seq and qRT-PCR for 8 core drought-responsive candidate genes across six comparison groups of maize *ZmEIL2* transgenic lines. **(A–C)** Comparisons between wild-type (WT) and *ZmEIL2*-overexpressing (*ZmEIL2*-OE) maize under normal (0 d), moderate drought (10 d), and severe drought (13 d) treatments, respectively. **(D–F)** Comparisons between wild-type (WT) and *ZmEIL2*-knockout (*ZmEIL2*-KO) maize under 0 d, 10 d, and 13 d drought treatments, respectively. The black dashed lines represent linear regression fitting curves, and r^2^ indicates the coefficient of determination for each pairwise correlation analysis. High r^2^ values (0.870–0.913) reveal strong consistency between transcriptome sequencing and qRT-PCR results, verifying the reliability of RNA-seq expression profiles.

## 3 Discussion

### 3.1 *ZmEIL2*, an *EIN3/EIL* family transcription factor, participates in drought resistance regulation and holds potential for molecular breeding in Maize

The *EIN3/EILs* protein family consists of core transcription factors within the ethylene signaling pathway and plays crucial roles in plant growth, development, and responses to environmental stresses. In Arabidopsis thaliana, *EIN3* and *EIL1* serve as the primary transcriptional regulators of ethylene responses; the ein3 eil1 double mutant exhibits complete insensitivity to ethylene, as evidenced by the absence of the “triple response” and increased susceptibility to pathogens. Functioning as a signal integration hub, the *EIN3/EIL1* transcription factor fine-tunes ethylene responses in a spatiotemporal and context-dependent manner (Dolgikh et al., 2019). In maize, a genome-wide identification has revealed nine ZmEIL genes, which exhibit distinct expression patterns across various tissues and under different stress conditions (Jyoti et al., 2021). Our study demonstrates that the overexpression of *ZmEIL2* significantly enhances drought tolerance in maize, while the knockout of this gene results in the opposite phenotype, indicating that *ZmEIL2* acts as a positive regulator of drought resistance. This finding aligns with the established role of the *EIN3/EIL* family as key regulators of ethylene signaling (Zhang and Dong, 2016).

By integrating phenotypic, physiological, hormonal, and transcriptomic data from this study, we identify *ZmEIL2* as a positive regulatory factor for drought tolerance in maize. This gene enhances drought resistance through various mechanisms, including the regulation of stomatal movement, coordination of hormonal homeostasis, accumulation of osmoprotectants, and reinforcement of antioxidant defenses. Importantly, overexpression of *ZmEIL2* improves drought tolerance without causing significant adverse effects on normal plant growth and development, thereby positioning it as a promising candidate gene for molecular breeding. Collectively, our findings offer valuable genetic resources and a theoretical foundation for the development of drought-resilient maize varieties.

### 3.2 *ZmEIL2* enhances drought tolerance in maize through multi-layered regulatory mechanisms

Under drought stress, plants employ various adaptive mechanisms, including stomatal regulation, hormonal signaling integration, osmotic adjustment, and antioxidant defense. Our study demonstrated that *ZmEIL2*-overexpressing lines exhibited significantly lower leaf water loss rates and higher relative water contents under drought conditions, accompanied by markedly reduced stomatal apertures. These findings indicate that *ZmEIL2* promotes stomatal closure to mitigate transpirational water loss. The role of ethylene in stomatal regulation is complex and multifaceted: exogenous ethylene can induce stomatal closure through the accumulation of ROS, the synthesis of nitric oxide and hydrogen sulfide, and the activation of SLAC1 (Hasan et al., 2024); conversely, it can inhibit ABA-induced stomatal closure (Tanaka et al., 2005). This duality likely depends on ETH concentration, treatment duration, and plant species. In maize, the calcium-dependent protein kinases *ZmCPK35* and *ZmCPK37* enhance drought tolerance by activating the guard-cell anion channel *ZmSLAC1* (Li X. et al., 2022), whereas the PP2C phosphatase *ZmPP84* (Guo et al., 2023) and *ZmGCT1/2* (Feng et al., 2025) negatively regulate drought tolerance by suppressing stomatal closure. Therefore, the positive effect of *ZmEIL2* overexpression on stomatal closure in our study may be mediated through the regulation of these stomatal movement-related genes via the EIN3/EIL ETH signaling pathway.

ABA is a central hormone in plant responses to drought. We observed significant alterations in both endogenous ETH and ABA levels in *ZmEIL2*-overexpressing lines subjected to drought stress, suggesting that *ZmEIL2* may coordinate these two major hormonal signaling pathways. In rice, *OsEIL1* regulates ETH-inhibited root elongation by activating YUC8 transcription and promoting auxin biosynthesis (Qin et al., 2017). Additionally, both *OsEIL1* and *OsEIL2* facilitate coleoptile elongation and seedling emergence by activating ROS-scavenging genes (Qiao et al., 2024). The interaction between ETH and ABA is particularly crucial for stomatal movement; ETH can delay ABA-induced stomatal closure (Tanaka et al., 2005), yet under certain conditions, it can also act synergistically with ABA to promote closure (Hasan et al., 2024). In our study, the overexpression of *ZmEIL2* enhanced stomatal closure while simultaneously increasing ABA accumulation. This finding appears to contradict the well-documented antagonistic role of ETH in ABA-induced stomatal closure, where ETH inhibits ABA signaling through the EIN2-EIN3-PP2C module (An et al., 2025). We propose several potential explanations for this apparent paradox. First, the overall enhancement of ABA signaling may surpass the inhibitory effect of ETH, as the dynamic equilibrium between ABA receptors and PP2Cs can be altered by sufficiently high levels of ABA (Müller, 2021). Second, members of the EIN3/EIL family exhibit functional diversification across species; thus, *ZmEIL2* may have distinct target specificity compared to AtEIN3 (Mao et al., 2022). Consequently, *ZmEIL2* may possess unique target gene specificity and regulatory properties relative to its *Arabidopsis* homolog *AtEIN3*. Consistently, EIL transcription factors have been shown to positively regulate stomatal closure and drought tolerance in several crop species, indicating a functional divergence from the Arabidopsis model (Ban et al., 2026). Third, the role of ETH in stomatal regulation is highly context-dependent. Under severe drought stress, ETH may transition from an inhibitor to a facilitator of ABA-mediated stomatal closure (Hasan et al., 2024). Collectively, these mechanisms may explain the enhanced stomatal closure observed in *ZmEIL2*-OE lines, despite elevated ETH levels. We propose that *ZmEIL2* may mitigate the potential inhibitory effect of ETH on stomatal closure by amplifying ABA signaling; alternatively, ETH may serve as a positive regulatory signal within this specific physiological context.

Drought stress induces excessive accumulation of ROS, leading to lipid peroxidation and membrane damage. Our data indicate that *ZmEIL2*-overexpressing lines exhibit significantly reduced levels of MDA and electrolyte leakage, alongside markedly increased proline content. This suggests that *ZmEIL2* effectively mitigates membrane damage and enhances osmotic adjustment. Proline is crucial for maintaining cellular turgor, protecting macromolecular structures, and regulating redox balance (Szabados and Savoure, 2010). In maize, several transcription factors enhance drought tolerance by modulating osmotic regulators: *ZmNAC49* reduces stomatal density (Xiang et al., 2021), *ZmNF-YA1* and *ZmNF-YB16* regulate stress-responsive gene expression (Yang et al., 2022), *ZmPTF1* promotes root development and ABA synthesis (Li et al., 2019), and natural variation in *ZmTIP1* enhances drought tolerance through root hair elongation (Zhang et al., 2020). The increased accumulation of proline in *ZmEIL2*-overexpressing lines may arise from direct regulation of proline biosynthesis genes or indirect activation of ABA-dependent osmotic responses via ETH signaling.

*ZmEIL2* overexpression significantly increased the activities of SOD, POD, and APX under drought stress. DAB and NBT staining revealed a marked reduction in ROS accumulation, indicating that *ZmEIL2* strengthens the antioxidant enzyme system to eliminate excess ROS. This observation aligns with the established role of ETH signaling in regulating antioxidants; ETH can induce ROS production while simultaneously activating antioxidant enzymes to maintain ROS homeostasis (Hasan et al., 2024). In maize, *ZmNAC84* enhances drought tolerance by directly upregulating *ZmSOD2* expression (Han et al., 2021). *ZmWRKY79* positively regulates tolerance through ABA biosynthesis (Gulzar et al., 2021). *ZmHsf28* functions through jasmonic acid and ABA signaling (Liu et al., 2023). *ZmEREB24* binds to the promoters of downstream target genes to improve tolerance (Ren et al., 2024). *ZmDof22* regulates stomatal movement and antioxidant enzyme activity (Cao et al., 2024). Lastly, *ZmC2H2-149* negatively regulates tolerance by repressing *ZmHSD1* (Liu et al., 2024). As a key transcription factor in the ETH signaling pathway, *ZmEIL2* likely enhances ROS scavenging by directly activating the expression of antioxidant enzyme genes.

Our findings collectively demonstrate that *ZmEIL2* enhances drought tolerance in maize through coordinated, multi-layered mechanisms, which include stomatal regulation, hormonal balance, osmotic adjustment, and antioxidant defense, while not significantly adversely affecting normal growth and development. Therefore, *ZmEIL2* serves as a promising candidate gene for molecular breeding efforts focused on improving drought resilience in maize.

### 3.3 The transcriptional network regulated by *ZmEIL2* and its association with known drought-resistant genes

To thoroughly investigate the molecular regulatory network underlying *ZmEIL2*-mediated drought resistance in maize, this study identified 120 core differentially expressed genes that consistently respond to drought across various drought gradients and genetic backgrounds. This identification was achieved through the intersection analysis of six groups of genotype-drought treatment differential genes. The functions of these genes encompass multiple levels, including transcriptional regulation, hormone metabolism, reactive oxygen species clearance, and cell wall remodeling, thereby confirming that *ZmEIL2* enhances maize’s drought tolerance through synergistic regulation across multiple pathways. Notably, the AP2/ERF family transcription factors *ZmERF115* (*Zm00001d016616*) and *ZmERF112* (*Zm00001d021089*) exhibit differential expression under drought stress. This family has been widely recognized for its role in regulating abiotic stress responses in maize. For example, *ZmERF21* positively regulates maize’s drought resistance via ETH, ABA, and calcium signaling pathways (Wang et al., 2022), while its rice homolog, *OsERF115*, enhances plant tolerance to both high temperature and drought stress by promoting proline synthesis (Park et al., 2021). The expression patterns observed in both overexpression and knockout lines suggest that these factors may be indirectly regulated by *ZmEIL2* or may operate through a feedback inhibition mechanism. The bZIP family transcription factor *ZmbZIP25* (*Zm00001d030995*) is positively regulated by *ZmEIL2* under severe drought conditions. This family constitutes a fundamental component of the ABA signaling pathway. Both *ZmbZIP72* (Ying et al., 2012) and *ZmbZIP4* (Ma et al., 2018) have been reported to enhance plant drought resistance through the ABA-dependent pathway. The regulation of root development and stress response gene expression implies that *ZmbZIP25* may function as a downstream factor in ABA signaling, contributing to the drought resistance regulatory network of *ZmEIL2*. The key enzyme genes *ZmAOS1* (*Zm00001d048021*) and *ZmOPR2* (*Zm00001d044906*), which are involved in jasmonic acid biosynthesis, were significantly upregulated in overexpression lines under drought conditions. Notably, *ZmOPR2* has been confirmed to mediate jasmonic acid metabolism and defense responses in maize under biological stress (Huang et al., 2023). Given the core regulatory role of jasmonic acid in abiotic stress, this suggests that *ZmEIL2* can synergistically enhance the response to drought stress by activating the jasmonic acid biosynthetic pathway. Additionally, the glutathione S-transferase encoding gene *ZmGSTU6* (*Zm00001d043795*) and the heat shock transcription factor *ZmHSF4* (*Zm00001d020714*) were upregulated in the overexpression line. *ZmGSTU6* mitigates oxidative damage and enhances cellular detoxification under drought conditions by catalyzing glutathione binding reactions (Göttlinger et al., 2026; Liu et al., 2022). In contrast, *ZmHSF4* plays a role in the regulatory mechanisms of cross-adaptation to high temperature and drought stress (Hayford et al., 2024; Eisenhauer et al., 2025; Gao et al., 2024). Together, these genes constitute the oxidative defense and stress cross-tolerance pathway downstream of *ZmEIL2*. In summary, the key functional components of these core drought response genes are substantiated by research on abiotic stress regulation. The differential expression patterns of these genes between *ZmEIL2* overexpression and knockout lines systematically demonstrate that *ZmEIL2* integrates multiple pathways, including ABA signaling, jasmonic acid metabolism, reactive oxygen species clearance, and molecular partner protection, thereby providing a molecular basis for the synergistic enhancement of drought tolerance in corn.

All phenotypic and mechanistic analyses in this study were conducted under controlled pot-culture conditions. While our findings elucidate the regulatory role of ZmEIL2 in drought stress responses at physiological and molecular levels, the inferences made may not completely represent the behavior of transgenic maize in intricate field settings. Additional comprehensive field trials are necessary to assess the impact of *ZmEIL2* on yield and agronomic characteristics under authentic drought conditions. Consequently, conducting field-based phenotyping of *ZmEIL2*-OE and *ZmEIL2*-KO lines will be a crucial avenue for subsequent research.

## 4 Materials and methods

### 4.1 Plant materials and stress treatments

The maize inbred line B104 was used as the genetic background in this study. Previously, overexpression and CRISPR/Cas9-mediated knockout transgenic lines of the *ZmEIL2* gene were successfully generated. RT-qPCR analysis was performed using gene-specific primers listed in **Supplementary Table 6**. RT-qPCR revealed a substantial upregulation of *ZmEIL2* expression in the homozygous overexpression lines OE1 (42.5-fold) and OE3 (71.1-fold) compared with the wild-type (P < 0.01, Student’s *t*-test) (**Supplementary Figure 1A**). For genotyping of knockout lines, target fragments were amplified with primers provided in **Supplementary Table 6**, and the homozygous mutation status was confirmed by Sanger sequencing. Both KO3 and KO4 exhibited clean single-peak chromatograms at the sgRNA target sites, verifying homozygous mutations. Specifically, KO3 carried a single cytosine (C) deletion at position 403 of the coding sequence, whereas KO4 harbored a single guanine (G) insertion at position 535. Both variants caused open-reading-frame frameshifts, resulting in premature translational termination and functional knockout of *ZmEIL2*.

Wild-type (WT), overexpressor (OE), and knockout (KO) seeds were sown in a nutrient soil and vermiculite mixture (2:1) within a growth chamber maintained at 25 ± 2 °C, with a 16/8 h light/dark photoperiod, 70% relative humidity, and light intensity of 300 μmol m^-2^ s^-1^. All pots were fully irrigated to field capacity before water withholding.

Drought stress was imposed via water withholding at the three-leaf stage, following a phenotype endpoint-driven strategy with two independent parallel assays. Severe wilting was uniformly defined as the treatment endpoint, characterized by curled, drooping leaves and collapsed plant stature: (1) WT and KO lines (KO3/KO4) were cultivated and treated in the same batch. Due to the impaired drought tolerance of KO lines, the predefined severe wilting endpoint was reached after 12 d of drought treatment; photographs were taken at this time point, and plants were subsequently rewatered. (2) WT and OE lines (OE1/OE3) were cultivated and treated in the same batch. Due to the enhanced drought tolerance of OE lines, 14 d of water withholding were required to reach the same wilting endpoint; photographs were taken at this time point, followed by rehydration. Recovery phenotypes were recorded 3 d after rewatering, and the survival rate was calculated for each genotype. Each assay included its own contemporaneous WT control, and only within-batch comparisons between each transgenic line and its matched WT were performed.

### 4.2 Measurement of water loss rate and RWC

At the three-leaf stage, the second leaves of WT, OE, and KO seedlings were excised and placed on filter paper at room temperature. Fresh weight was recorded immediately (FW_0_) and subsequently at 0 h, 6 h, 12 h, 24 h, and 48 h (FW_t_), while photographs should be taken at various processing time points. The water loss rate was calculated using the formula: water loss (%) = [(FW_0_ − FW_t_)/FW_0_] × 100 (Li et al., 2017). For the determination of relative water content (RWC), leaves were first weighed to obtain fresh weight, then soaked in distilled water for 12 h to achieve saturated fresh weight, and finally dried at 70 °C for 12 h to ascertain dry weight. RWC was calculated as follows: RWC (%) = [(fresh weight − dry weight)/(saturated fresh weight − dry weight)] × 100. Three biological replicates were performed.

### 4.3 Observation of stomatal morphology and quantitative measurement of opening degree

maize leaves at the three-leaf and one-heart stage were rinsed with distilled water, dried with filter paper, and immersed in stomatal opening buffer solution (10 mM KCl, 50 mM CaCl_2_, 10 mM Tris, pH 5.6) for a 2 h dark treatment followed by a 3 h light treatment. Subsequently, the leaves underwent dehydration treatments for 0 min, 15 min, and 20 min. A thin layer of clear nail polish was applied to the epidermis between the veins and the margin of the second leaf to create an impression. After drying, the epidermal film was carefully removed and mounted on a glass slide for observation under an optical microscope (20×). Each treatment was replicated three times, with fifty stomata randomly chosen for measurement in each replicate. Stomatal dimensions (length and width) were quantified using ImageJ software. Stomatal aperture was determined as the width-to-length ratio, and statistical analysis was performed.

### 4.4 Determination of ETH and ABA content

At 0 d, 10 d, and 13 d of drought treatment, the leaves of WT, OE and KO lines were taken respectively for the determination of ETH and ABA contents.

ETH determination: Approximately 10 g of fresh leaves should be placed in a conical flask, which is then sealed with a rubber stopper. The flask must be incubated in a shaker at 30 °C for 20 h to facilitate the complete release of ETH. A volume of 30 mL of headspace gas is collected into a sampling bag using the drainage gas collection method and subsequently analyzed using a gas chromatograph (GC). The detection conditions are as follows: injection port at 60 °C, detector at 150 °C, and column temperature at 60 °C. Sample injection and detection occur 30 min after the baseline stabilizes. The formula for calculating ETH concentration is as follows: ETH concentration (μL/L) = sample peak height × standard ETH concentration/standard peak height.

ABA determination: Begin by taking 0.5 g of fresh leaves and grinding them into a powder using liquid nitrogen. Next, add 5 mL of an isopropyl alcohol-hydrochloric acid extract and shake the mixture at 4 °C in the dark for 30 min. Subsequently, introduce 10 mL of dichloromethane and continue shaking at 4 °C for an additional 30 min. Centrifuge the mixture at 13,000 rpm for 5 min at 4 °C, then carefully aspirate the underlying organic phase. After drying under nitrogen, redissolve the residue in 350 μL of a methanol solution containing 0.1% formic acid. Finally, filter the solution through a 0.45 μm organic filter membrane to prepare it for analysis.

### 4.5 Determination of cell membrane damage and osmotic regulatory substances

The contents of MDA and Pro were measured in transgenic and wild-type lines subjected to natural drought for 0 d, 10 d, and 13 d. The contents of MDA and Pro were determined using the MDA Assay Kit and the Proline Assay Kit (JC DTECT, Nanjing, China). The REC of the leaves was assessed using the immersion method. The electrical conductivity of the solution was measured before and after boiling with a conductivity meter to calculate the relative electrical conductivity.

### 4.6 Detection of ROS and determination of antioxidant enzyme activity

Leaves of WT, OE and KO lines treated with drought for 0 d, 10 d, and 13 d were taken for the following tests.

Histochemical staining: The leaves were immersed in 1 mg·mL^-1^ DAB solution to detect H_2_O_2_ and in 0.5 mg·mL^-1^ NBT solution to detect O_2_^-·^. They were then incubated at room temperature in the dark for either 10 h or 12 h, decolorized with 95% ethanol for 10 min, and subsequently photographed for documentation.

Quantitative determination: The H_2_O_2_ assay kit, O_2_^-·^ assay kit, SOD assay kit, POD assay kit, and APX assay kit (JC DTECT, Nanjing) were employed to quantify the levels of H_2_O_2_ and O_2_^-·^, as well as to assess the activities of SOD, POD, and APX, respectively.

### 4.7 Transcriptome sequencing and analysis

#### 4.7.1 Plant material and treatment for RNA-seq

Wild-type (B104), *ZmEIL2*-OE, and *ZmEIL2*-KO lines were used for transcriptome sequencing. Uniform and healthy seeds were surface-sterilized with 75% ethanol for 5 min and rinsed 3–5 times with sterile water. Seeds were sown in a soil:vermiculite (2:1) mixture and grown in a growth chamber at 25 °C. At the three-leaf stage, uniform seedlings were subjected to natural drought treatment for 10 d and 13 d. Leaf tissues were harvested from the same region of the second functional leaf of each plant, with three independent biological replicates. All sample preparation and transcriptome sequencing were performed by BGI Genomics Co., Ltd. (Shenzhen, China).

#### 4.7.2 RNA-seq data analysis

Raw sequencing reads were quality-filtered to obtain clean reads for downstream analysis. Clean reads were aligned to the maize B73 RefGen_v4 reference genome using HISAT2 (Kim et al., 2015), and the reference gene set was aligned using Bowtie2 (Langmead and Salzberg, 2012). Gene expression levels were quantified as fragments per kilobase of transcript per million mapped reads (FPKM) using RSEM (Li and Dewey, 2011). Differential expression analysis between groups was performed using DESeq2 (Love et al., 2014). Genes with Q-value ≤ 0.05 or FDR ≤ 0.001 were defined as differentially expressed genes (DEGs).

#### 4.7.3 DEG screening and GO functional annotation

Based on the maize B73 V4 reference genome, read counts were normalized using DESeq2. DEGs were screened with thresholds of |log_2_(fold change)| ≥ 1 and FDR < 0.05. GO functional enrichment analysis was subsequently performed to annotate the biological functions of the identified DEGs.

#### 4.7.4 RNA extraction and RT-qPCR analysis

Total RNA was extracted from sampled leaves using TRIzol reagent (Invitrogen, USA) according to the manufacturer’s instructions. First-strand cDNA was synthesized from extracted RNA using the PrimeScript™ RT reagent Kit with gDNA Eraser (TaKaRa, Dalian, China). RT-qPCR was performed using TB Green^®^ Premix Ex Taq™ II (TaKaRa, Dalian, China) on a LightCycler 96 real-time PCR system (Roche Diagnostics, Shanghai, China). Relative gene expression was calculated using the 2^-ΔΔCt^ method (Livak and Schmittgen, 2001), with *ZmActin1* as the internal control. Three biological and three technical replicates were performed for each assay. Specific primer sequences are listed in **Supplementary Table S5**.

#### 4.7.5 qRT-PCR validation of RNA-seq data

To validate the reliability of the RNA-seq data, eight randomly selected DEGs with consistent expression trends were subjected to qRT-PCR analysis. Six pairwise comparison groups were established across three genotypes (WT, KO, OE) and three treatments (0 d, 10 d, 13 d): WT vs KO 0 d, WT vs KO 10 d, WT vs KO 13 d, WT vs OE 0 d, WT vs OE 10 d, and WT vs OE 13 d. Total RNA from the same tissue samples used for RNA-seq was reverse-transcribed to cDNA. Fold changes (FC) in gene expression were calculated for each comparison group from both RNA-seq and qRT-PCR data and converted to -log_2_(FC) values. Scatter plots were generated with RNA-seq -log_2_(FC) on the x-axis and qRT-PCR -log_2_(FC) on the y-axis for each comparison group. Linear regression analysis was performed to evaluate the correlation between the two datasets, with the coefficient of determination (R^²^) calculated accordingly.

### 4.8 Statistical analysis

All experimental data were compiled and processed using Microsoft Excel 2019. Graphs were generated using Origin 2024 (OriginLab, Northampton, MA, USA). Statistical analyses were performed using SPSS 27.0 (IBM, Armonk, NY, USA) with Duncan’s multiple range test for mean comparisons. Significance levels were set at P < 0.05 for significant differences and *P < 0.01 for highly significant differences. All data are presented as means ± standard deviation (SD) from at least three independent biological replicates. Figure assembly and image export were performed using Adobe Illustrator 2025 (Adobe Inc., San Jose, CA, USA).

## Data Availability

The datasets presented in this study can be found in online repositories. The names of the repository/repositories and accession number(s) can be found below: https://www.ncbi.nlm.nih.gov/, PRJNA1493269.
